# Tree Leaf Bacterial Community Structure and Diversity Differ along a Gradient of Urban Intensity

**DOI:** 10.1128/mSystems.00087-17

**Published:** 2017-12-05

**Authors:** Isabelle Laforest-Lapointe, Christian Messier, Steven W. Kembel

**Affiliations:** a Département des sciences biologiques, Université du Québec à Montréal, Montréal, Québec, Canada; b Centre d’étude de la forêt, Université du Québec à Montréal, Montréal, Québec, Canada; c Institut des Sciences de la Forêt tempérée, Université du Québec en Outaouais, Ripon, Québec, Canada; Lawrence Berkeley National Laboratory

**Keywords:** biodiversity, bioindicators, microbial communities, microbial ecology, phyllosphere-inhabiting microbes, plant-microbe interactions, urban gradient, urban microbiome

## Abstract

Tree leaf-associated microbiota have been studied in natural ecosystems but less so in urban settings, where anthropogenic pressures on trees could impact microbial communities and modify their interaction with their hosts. Additionally, trees act as vectors spreading bacterial cells in the air in urban environments due to the density of microbial cells on aerial plant surfaces. Characterizing tree leaf bacterial communities along an urban gradient is thus key to understand the impact of anthropogenic pressures on urban tree-bacterium interactions and on the overall urban microbiome. In this study, we aimed (i) to characterize phyllosphere bacterial communities of seven tree species in urban environments and (ii) to describe the changes in tree phyllosphere bacterial community structure and diversity along a gradient of increasing urban intensity and at two degrees of tree isolation. Our results indicate that, as anthropogenic pressures increase, urban leaf bacterial communities show a reduction in the abundance of the dominant class in the natural plant microbiome, the *Alphaproteobacteria*. Our work in the urban environment here reveals that the structures of leaf bacterial communities differ along the gradient of urban intensity. The diversity of phyllosphere microbial communities increases at higher urban intensity, also displaying a greater number and variety of associated indicator taxa than the low and medium urban gradient sites. In conclusion, we find that urban environments influence tree bacterial community composition, and our results suggest that feedback between human activity and plant microbiomes could shape urban microbiomes.

**IMPORTANCE** In natural forests, tree leaf surfaces host diverse bacterial communities whose structure and composition are primarily driven by host species identity. Tree leaf bacterial diversity has also been shown to influence tree community productivity, a key function of terrestrial ecosystems. However, most urban microbiome studies have focused on the built environment, improving our understanding of indoor microbial communities but leaving much to be understood, especially in the nonbuilt microbiome. Here, we provide the first multiple-species comparison of tree phyllosphere bacterial structures and diversity along a gradient of urban intensity. We demonstrate that urban trees possess characteristic bacterial communities that differ from those seen with trees in nonurban environments, with microbial community structure on trees influenced by host species identity but also by the gradient of urban intensity and by the degree of isolation from other trees. Our results suggest that feedback between human activity and plant microbiomes could shape urban microbiomes.

## Abstract (precis)

In natural forests, tree leaf surfaces host diverse bacterial communities whose structure and composition are primarily driven by host species identity. Tree leaf bacterial diversity has also been shown to influence tree community productivity, a key function of terrestrial ecosystems. However, most urban microbiome studies have focused on the built environment, improving our understanding of indoor microbial communities but leaving much to be understood, especially in the nonbuilt microbiome. Here, we provide the first multiple-species comparison of tree phyllosphere bacterial structures and diversity along a gradient of urban intensity. We demonstrate that urban trees possess characteristic bacterial communities that differ from those seen with trees in nonurban environments, with microbial community structure on trees influenced by host species identity but also by the gradient of urban intensity and by the degree of isolation from other trees. Our results suggest that feedback between human activity and plant microbiomes could shape urban microbiomes.

## INTRODUCTION

While the human population in urban centers is estimated to increase by 2 to 4 billion this century (1), the focus of public health research is expanding from the benefits of urban plant communities (air quality, physical activity, social cohesion, and stress reduction) (2) to include the potential roles of the urban microbiota. The positive influence of urban vegetation on human physical health has been demonstrated many times (3, 4), but it could also play an unexpected role in the microbial communities that they support and in their contribution to urban biodiversity. Studies using high-throughput sequencing techniques are rapidly improving our understanding of the urban microbiome, defined as the ensemble of microbial organisms residing or transiting in the urban environment (5). Land use type (e.g., forest, rural, urban) has been shown to impact airborne microbial communities (6, 7), and recent work has demonstrated that the local vegetation influences the airborne bacterial community composition and abundance in urban settings (8) and natural settings (9). Most urban microbiome research has been done on the built environment (indoor space of human-built structure) (but see references 8, 10, and 11), improving our understanding of urban microbial communities but leaving much to be defined, especially in the nonbuilt environmental microbiome. In addition, the surrounding plant community has been suggested to influence the microbial community of key buildings frequented by the human population (i.e., hospitals, schools, and homes) (12–14). Therefore, characterizing the assembly and dynamics of the urban plant microbiome is crucial to ascertain the influence of higher anthropogenic pressures on these microbial communities and to describe their potential contribution to the urban microbiome.

The phyllosphere, defined as the leaf surfaces of plants, is estimated to have a surface area of 4 × 10^8^ km^2^ across the globe (15) and thus represents a major potential source of local microbial organisms (16, 17). In addition to its contribution to the urban microbiome, the canopy of urban trees provides a variety of services such as reducing local temperature, limiting water runoff, and increasing air quality (18). Recent research on the phyllosphere has found host species identity to be the key driver of leaf microbial community structure in both tropical ecosystems (19–23) and temperate ecosystems (24–28). However, to our knowledge few studies have described the changes in plant-associated microbiota from the natural environment to urban environments (but see reference 29 for bacterial communities on *Hedera* sp. and reference 30 for fungal communities on *Quercus macrocarpa*), leaving much to be learned on how the plant microbiome changes with increasing anthropogenic pressures. In this report, we focus on tree phyllosphere bacterial communities along a gradient of urban intensity to quantify the similarities and differences in community structure in the urban plant microbiome.

In the urban ecology literature, many different study-specific methodologies have been employed to estimate the intensity of human influence in an area, reducing the potential to compare results between experiments. In an attempt to standardize our work, we use the well-described index by Sanderson et al. (31) which has also been employed previously in a similar plant community urban gradient study (32). This index allows the evaluation of the anthropogenic impact on terrestrial ecosystems through multiple parameters, including population density, built-up areas, road access, landscape use, and electric power infrastructure, using nine public data sets (31). This index focuses on direct measures of human infrastructures and population, which are suggested to have the greatest and most direct effect on terrestrial ecosystems.

The urban environment differs strikingly from the natural forest environment mainly through an increase in the biotic and abiotic stresses caused directly and indirectly by anthropogenic activities. The increase in anthropogenic pressures in urban areas reduces tree fitness and longevity (33) and could modify many important functional traits (32). Numerous studies have shown that anthropogenic activities increase levels of leaf macronutrients (nitrogen, potassium, sulfur), micronutrients (boron, manganese, selenium), and trace elements (cadmium, lead, zinc) for urban trees (30, 34, 35). Higher temperatures in the urban environment influence vegetation phenology (36–38) and are intensified by city growth and global warming (39). The urban heat island phenomenon (40) results from the increase in the levels of nonpenetrating surfaces (41) and the decrease in the levels of vegetation cover (42) in cities. Thermal accumulation could drive enzymatic processes, affecting microbial communities directly, and could also increase the presence of insect ectotherms (43), which are known disease vectors (44). This increase in insect pest abundance in urban areas (45, 46) could also be intensified by changes in host plant quality and natural enemy efficiency (47). In addition to these stresses, urban trees frequently suffer from limited access to water and nutrients (48, 49), root development limitation (see reference 50 for a review), and photosynthetic biomass loss and tree lesions (51). These stresses have been shown to affect plant survival (52–54) and to induce numerous physiological responses, a phenomenon that could cause profound changes in urban tree leaf microbial communities. Therefore, urban biotic and abiotic conditions could provoke changes in the tree phyllosphere microbial community, potentially impacting host fitness and modifying the local pool of urban microbial organisms. However, little is known about the identity and dynamics of the urban tree phyllosphere.

To improve our understanding of the urban tree microbiome, we aimed (i) to characterize the bacterial communities present in tree phyllosphere bacterial communities of the urban environment and (ii) to describe the changes in tree phyllosphere bacterial community structure and diversity along a gradient of increasing urban intensity and at two degrees of tree isolation. Our hypotheses are (i) that the increasing urban intensity influences the abundance of the main taxonomical groups of bacteria usually described in the natural temperate forest phyllosphere literature and (ii) that the amount of stresses on trees imposed by higher anthropogenic pressures in urban agglomerations (nutrient enrichment, heat increase, physical stress, etc.) impacts phyllosphere bacterial community structure and reduces its diversity from low urban intensity to higher urban intensity settings. While previous urban microbiome studies have focused on air and built environment microorganisms (but see references 8, 10, and 11), our report provides key information on the urban plant-associated microbiota at different levels of urban intensity and offers new explanatory paths to better understand the gap between natural and urban environment microbiomes.

## RESULTS

**FIG 1:**
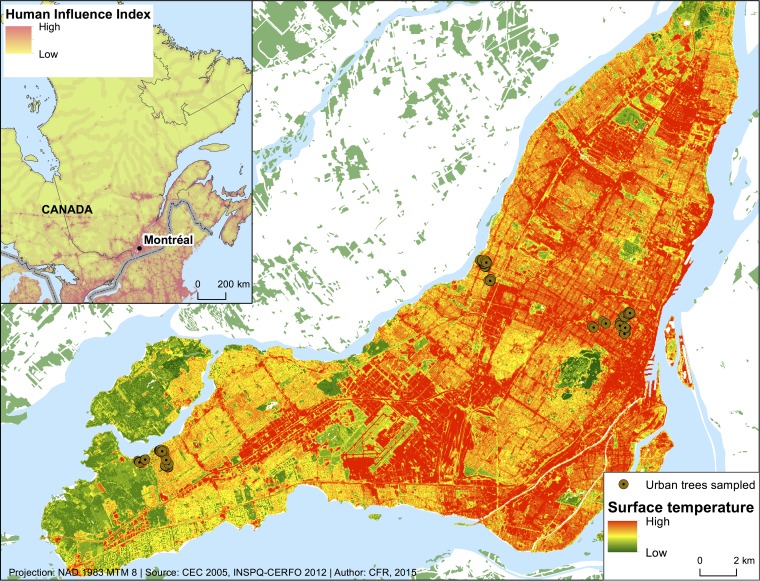
Locations of trees sampled along an urban gradient (three intensities: low, medium, and high) on the Island of Montreal, Canada. Temperature is used as a layover in this figure as an example of anthropogenic pressures along the gradient of intensity. The map was created using data from references 90, 91, and 92.

To characterize the tree phyllosphere microbiome found in urban settings, we sampled seven tree species (*Acer platanoides*, *Acer rubrum*, *Acer saccharum*, *Celtis occidentalis*, *Fraxinus americana*, *Fraxinus pennsylvanica*, and *Picea glauca*) at three sites in Montreal, Canada, along a gradient of increasing anthropogenic pressures. This urban intensity gradient was based on a composite index of human influence (IHI [31]) as employed by Nock et al. (32) where plots are classified in three categories: low, medium, and high anthropogenic pressures (Fig. 1). At each site, six individuals per tree species were randomly selected from the public district database to be sampled: three in parks (with tree neighbors and close plant community) and three in streets (no close tree neighbors and no close plant community) (see Table S1 in the supplemental material).

10.1128/mSystems.00087-17.1 TABLE S1  Description of the three study sites sampled during summer of 2014. Download TABLE S1, DOCX file, 0.02 MB. Copyright © 2017 Laforest-Lapointe et al. 2017 Laforest-Lapointe et al. This content is distributed under the terms of the Creative Commons Attribution 4.0 International license.

Our data set contained 8,671 operational taxonomic units (OTUs) with 3,634 to 32,060 sequences per sample for a total of 1,755,757 quality sequences from 126 samples. We rarefied the samples to 3,000 sequences each, with 18 samples excluded due to insufficient sequence reads as a result of extraction or sequencing errors.

**FIG 2:**
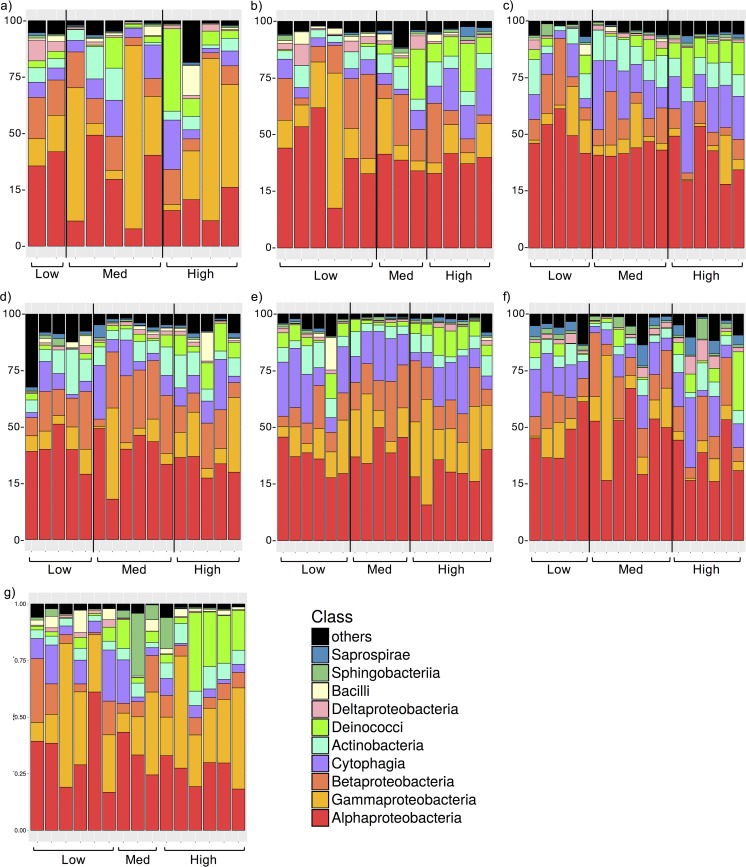
Relative abundance (%) of sequences from bacterial classes in the phyllosphere microbiome. Data represent the class community composition of (a) *Acer platanoides*; (b) *Acer rubrum*; (c) *Acer saccharum*; (d) *Fraxinus americana*; (e) *Fraxinus pennsylvanica*; (f) *Picea glauca*; and (g) *Celtis occidentalis*. Med, medium.

**FIG 3:**
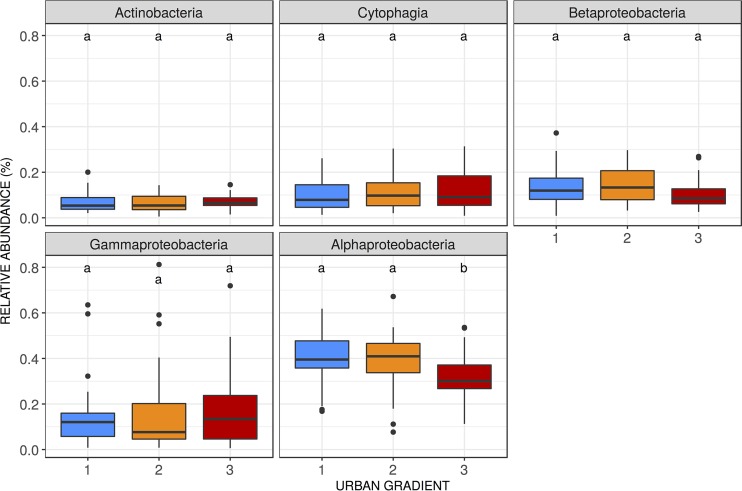
Relative abundances of the five most abundant classes along the urban gradient. Letters indicate the groups identified through a *post hoc* test of Tukey multiple comparisons of means at a 95% family-wise confidence level.

Our results show that leaf bacterial community composition varies among individual trees, among tree species, and along the urban gradient (Fig. 2). Along the urban gradient, phyllosphere bacterial communities were dominated by *Alphaproteobacteria*, averaging 40.8%, 39.4%, and 31.9% of sequences in communities from settings of low, medium, and high urban intensity, respectively. The five most abundant bacterial classes in the urban phyllosphere were *Alphaproteobacteria*, *Gammaproteobacteria*, *Betaproteobacteria*, *Cytophagia*, and *Actinobacteria*. Only the members of the class *Alphaproteobacteria* displayed a significant change in relative abundance along the urban gradient (*post hoc* Tukey’s tests on analysis of variance [ANOVA]), with differences of 3.4% and 2.0% comparing the high-intensity sites to the low-intensity and medium-intensity sites, respectively (*F* = 6.7, *P* < 0.005) (Fig. 3).

**FIG 4:**
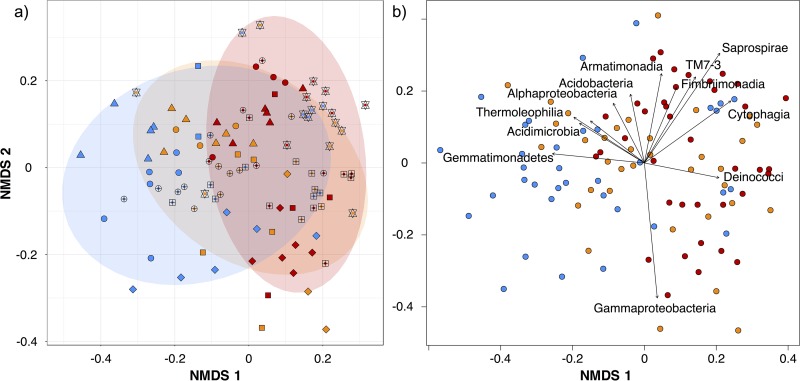
Nonmetric multidimensional scaling (NMDS) ordination of variation in bacterial community structure of tree phyllosphere along a gradient of urban intensity. Data represent ordination based on Bray-Curtis distances among 108 samples. Samples (points) are colored based on the urban gradient (blue for low intensity, orange for medium intensity, and red for high intensity). In panel a, shapes represent host species identity (squares for *Acer platanoides*; circles for *Acer rubrum*; triangles for *Acer saccharum*; diamonds for *Celtis occidentalis*; sun crosses for *Fraxinus Americana*; squares with cross for *Fraxinus pennsylvanica*; stars for *Picea glauca*); ellipses indicate 1 standard deviation confidence intervals around samples from urban gradient intensity. In panel b, arrows represent the significant (*P* < 0.001) correlations between NMDS axes versus the relative abundances of bacterial classes in communities.

We quantified the relative influences of multiple drivers on leaf bacterial community taxonomic and phylogenetic structure by conducting a permutational multivariate analysis of variance (PERMANOVA [55]) on Bray-Curtis dissimilarities and weighted Unifrac (56) distances among samples (Table 1). We compared the relative influences of multiple drivers of leaf bacterial community composition along the gradient of urban intensity: host species identity (*Acer platanoides*, *Acer rubrum*, *Acer saccharum*, *Celtis occidentalis*, *Fraxinus americana*, *Fraxinus pennsylvanica*, and *Picea glauca*), tree isolation (park or street), and urban intensity (low, medium, and high). Because the two models yielded highly similar results in terms of significant variables and explanatory power, here we present only the results of analyses based on Bray-Curtis dissimilarities. We illustrated bacterial community taxonomic structure patterns by performing a nonmetric multidimensional scaling (NMDS) ordination of Bray-Curtis dissimilarities (Fig. 4). The strongest driver of urban phyllosphere community structure was host species identity (*R*^2^ = 19.4%, *P* = 0.001; PERMANOVA on Bray-Curtis distances; Table 1). Urban intensity (*R*^2^ = 6.1%, *P* = 0.001) and tree isolation (*R*^2^ = 1.8%, *P* = 0.001) were both weaker but significant drivers of leaf community structure. All second-level interactions were significant, the strongest being the interaction between urban intensity and host species identity (*R*^2^ = 12.1%, *P* = 0.001), and the third-level interaction was also significant (R^2^ = 8.0%, *P* = 0.017). At the class taxonomic level, we found multiple significant correlations among the relative abundances of various bacterial groups with the two axes of the NMDS (Fig. 4b), including *Gemmatimonadetes*, *Deinococci*, *Acidomicrobia*, and *Thermoleophilia* with NMDS axis 1 and *Gammaproteobacteria*, *Acidobacteria*, *Alphaproteobacteria*, *Armatimonadia*, *Saprospirae*, TM7-3, *Fimbriimonadia*, and *Cytophagia* with NMDS axis 2. Because of the significance of the second- and third-level interactions, we quantified how each of the host tree species bacterial community structure was explained by the urban gradient and tree isolation by building individual host species models (Table 2). Urban intensity had the strongest explanatory power for the leaf bacterial communities of *A. rubrum* (*R*^2^ = 32.1%, *P* = 0.003), followed by *C. occidentalis* (*R*^2^ = 25.4%, *P* < 0.001), *F. americana* (*R*^2^ = 24.3%, *P* < 0.001), and *F. pennsylvanica* (*R*^2^ = 19.1%, *P* = 0.007). The effect of urban intensity was not significant for *A. saccharum* (*R*^2^ = 23.8.1%, *P* = 0.102) and was only marginally significant for *P. glauca* (*R*^2^ = 14.7%, *P* = 0.078). Since host species identity was the main driver of leaf bacterial community structure across the urban gradient, we built a model accounting for phylogenetic relatedness between host tree species to identify the explanatory power of different phylogenetic levels. The angiosperm/gymnosperm level explained 4.5% of the total variation in phyllosphere bacterial community structure, whereas the genus and species levels explained, respectively, 7.2% and 8.3% of the variation.

**TABLE 1:** Bacterial community structure explained by host species identity, urban intensity, and tree isolation (street or park) and their interactions^a^

Variable^b^	Bray-Curtis dissimilarity			Weighted Unifrac distance		
	*F* value	*R*^2^	Pr(>*F*)	*F* value	*R*^2^	Pr(>*F*)
1st level						
Host species identity	4.97	19.38	0.001	5.03	19.73	0.001
Urban intensity	4.66	6.05	0.001	5.07	6.63	0.001
Tree isolation	2.78	1.81	0.001	2.92	1.91	0.009
2nd level						
Urban intensity*						
Tree isolation	1.33	1.73	0.056	NS	NS	NS
Host species*						
Tree isolation	1.74	6.77	0.001	1.77	6.96	0.004
Urban intensity*						
Host species	1.55	12.10	0.001	1.49	11.71	0.005
3rd level						
Urban intensity*						
Host species*	1.23	7.98	0.017	NS	NS	NS
Tree isolation						

a Data represent results of PERMANOVA analysis of Bray-Curtis dissimilarities and weighted Unifrac distances. The models explained, respectively, 56% and 47% of the variation in bacterial taxonomical and phylogenetic community structure. Pr(>*F*), *P* value; NS, not significant.

b An asterisk represents interaction between variables.

**TABLE 2:** Bacterial community structure of individual host species explained by host urban intensity and tree isolation (street or park) and their interaction^a^

Host species	Urban intensity		Tree isolation		Urban intensity × tree isolation		Total *R*^2^ (%)
	*R*^2^ (%)	*P*	*R*^2^ (%)	*P*	*R*^2^ (%)	*P*	
*A. platanoides*	NS		16.0%	0.030	NS		16
*A. rubrum*	32.1	0.001	NS		10.3	0.034	42.4
*A. saccharum*	24.2	0.001	9.2	0.012	NS		33.4
*C. occidentalis*	25.4	0.001	13.3	0.003	NS		38.7
*F. americana*	24.3	0.001	NS		NS		24.3
*F. pennsylvanica*	19.1	0.009	NS		NS		19.1
*P. glauca*	NS		11.3	0.010	NS		11.3

a Data represent results of PERMANOVA analysis of Bray-Curtis dissimilarities.

**FIG 5:**
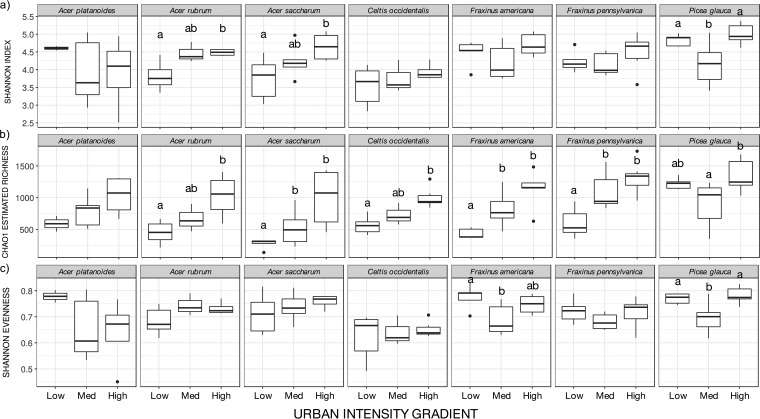
Community diversity indices of phyllosphere bacterial communities for different host species along the urban gradient. (a) Shannon diversity. (b) Chao 1 estimated richness. (c) Shannon evenness.

To further describe the changes in leaf bacterial community structure and diversity, we built a linear model to measure the relative influences of host species identity, urban intensity, and tree isolation on tree leaf bacterial alpha-diversity (Shannon diversity index). We compared the strengths of the variables in the best model by an ANOVA, which included urban intensity (*F* = 7.067, *P* = 0.001) and host species (*F* = 5.901, *P* = <0.001) as well as their interaction (*F* = 1.961, *P* = 0.037). Comparing the leaf bacterial alpha-diversity levels across sites, the highest level of urban intensity exhibited greater leaf bacterial alpha-diversity (mean = 4.5; Shannon index) than the low urban intensity (mean = 4.1, *P* = 0.004, *post hoc* Tukey’s test on ANOVA) and the medium urban intensity (mean = 4.1, *P* = 0.005, *post hoc* Tukey’s test on ANOVA). Since the interaction between the urban intensity and host species identity was significant, we computed per-host species alpha-diversity indices (Chao 1 and Shannon diversity), as well as community evenness (Shannon evenness) (Fig. 5). All species but *A. platanoides* showed an increase in estimated Chao 1 richness along the urban gradient, whereas only *A. rubrum* and *A. saccharum* demonstrated an increase in the Shannon diversity index value with increased urban intensity. Community evenness fluctuated across sites only for *F. americana* and *P. glauca*, which displayed a loss in community evenness at the medium level of the urban gradient.

**FIG 6:**
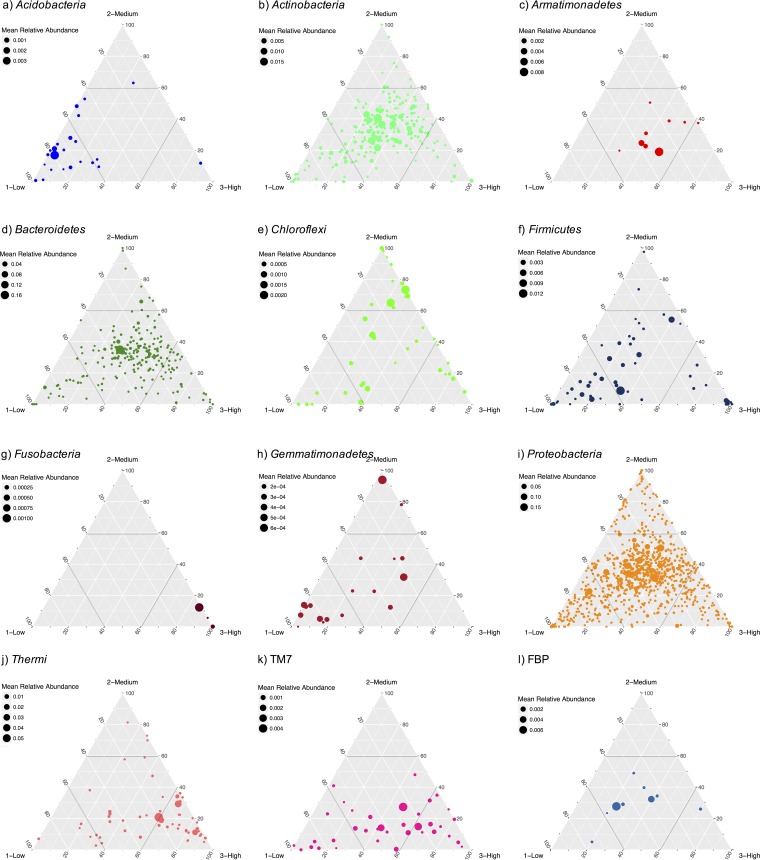
Ternary plots of per-site mean relative abundances of OTUs. Panels represent the 12 most abundant phyla. Each point represents an OTU, and its position indicates the proportion of its relative abundance at the different sites along the urban gradient. Points closer to the ternary plot corners indicate that a greater proportion of the total relative abundance of this OTU was found in this particular environment. Point colors indicate the phylum of the OTU. The lines inside the ternary plot indicate the 60% level of relative abundance of each of the sites. Only the 275 OTUs with a per-site mean relative abundance of more than 0.1% are shown.

In order to identify significant indicator species in the leaf microbiome of each of the sites of the urban gradient, we estimated the indicator values for each OTU (57). Following the methodology presented by Wilhelm et al. (58), OTUs with an indicator value higher than 0.3 and a *P* value of <0.05 were classified as indicator species (Table S2). We found 21 indicators associated with the low level of the urban gradient (including OTUs from the orders *Rhizobiales*, *Sphingomonadales*, *Rhodospirillales*, and *Burkholderiales*), 9 associated with the medium level (including OTUs from the orders *Actinomycetales*, *Rhodospirillales*, and *Sphingomonadales*), and 37 indicators associated with the high level (including OTUs from the orders *Cytophagales*, *Deinococcales*, *Rhodospirillales*, and *Actinomycetales*). In addition, we built a three-axis ternary plot (Fig. 6) for the 12 most abundant phyla to visualize the relative proportions of OTUs at the three sites along the urban gradient. Each of OTUs was fitted in an *x-y-z* coordinate system, with each corner of the triangle representing a site along the urban gradient. The ternary plot showed that many OTUs were present in similar proportions at the three sites of the urban gradient but that some were relatively more abundant at a specific site (Fig. 6). In agreement with the indicator analysis, a greater number of OTUs showed an association with either the low level or high level of the urban gradient than with the medium level.

10.1128/mSystems.00087-17.2 TABLE S2  Significant associations between bacterial OTUs and sites along the urban gradient detected by indicator species analysis (57). Panels represent (a) low (Pierrefonds), (b) medium (Ahuntsic), and (c) high (Mont-Royal) anthropogenic pressures. Download TABLE S2, DOCX file, 0.2 MB. Copyright © 2017 Laforest-Lapointe et al. 2017 Laforest-Lapointe et al. This content is distributed under the terms of the Creative Commons Attribution 4.0 International license.

## DISCUSSION

In this report, we describe the tree phyllosphere bacterial communities at three sites along a gradient of urban intensity (based on a planet-wide index of human influence; 31) and two levels of tree isolation among seven different tree species. In agreement with previous studies of the natural tree phyllosphere, our results show that the composition of tree leaf bacterial communities of the urban environment is determined mainly by the identity of the host species. However, we confirmed our first hypothesis, i.e., that urban intensity and, to a lesser extent, tree isolation also influence the identity of bioindicator taxa, community composition, and diversity. While the results presented in this work are new and informative for future urban plant microbiome studies, we acknowledge that our study did not describe leaf bacterial load, a factor that could reveal density-dependent effects on population structure in the phyllosphere. We suggest that future studies could address these issues using measurements of microbial load through quantitative PCR (qPCR) performed on extracted DNA (i.e., 16S), cell counting, and culture-based approaches (59). Nonetheless, in the context of the urban microbiome, this work provides an unprecedented description of urban plant-associated microbiomes, providing key information for future studies of the impact of urban conditions on leaf microbial communities as well as on the overall urban microbiome. In addition, studying the urban plant microbiome increases our knowledge of the potential sources of the air and built-environment microbiome, which offers great insights for the eventual management of the urban microbiome. Our results could also impact the agriculture and the food industry, where research effort is now being put into designing biological control agents as a means to replace common pest-management interventions that have a strong negative impact on human and environment health (i.e., pesticides). Recent studies showcase successful biocontrol examples with both bacteria (60) and fungi (61). However, our work shows that the plant leaf microbiome is a function of local environmental conditions; therefore, future studies could benefit from considering this factor while designing leaf microflora biocontrol interventions.

In previous studies of tree phyllosphere microbial communities in natural environments, host species identity had often been found to be the strongest determinant of community structure (20, 24, 26). Here, our results also show that host species identity is a stronger driver of leaf bacterial community structure than urban intensity or tree isolation (Table 1; Fig. 4). Although host tree species identity is the main determinant of leaf bacterial community structure, this driver explained 20% of the variation. Therefore, we cannot assume that controlling the urban forest composition would allow us to manage the entire local urban microbiome. The remaining 80% of the variation could be linked to other local (random dispersal, site history, control from other microorganisms, animal and insect vectors), regional (air contamination), and national phenomena such as large-scale atmospheric air movements (62).

In accordance with previous descriptions of the tree phyllosphere (20, 26–28, 63), the urban phyllosphere bacterial communities are dominated by the *Alphaproteobacteria* (Fig. 2). However, our results demonstrate that the abiotic and biotic changes induced by the urban environment reduced the relative abundance of this bacterial taxonomic group (*Alphaproteobacteria*; Fig. 3) and enriched or depleted the relative abundances of many specific OTUs (Fig. 6). Similar effects on the phyllosphere of ivy (*Hedera* sp.) have been found by Smets et al. (29), who showed shifts in leaf bacterial communities between nonurban and urban sites in relation to atmospheric contamination. Jumpponen and Jones (30) also showed that tree phyllosphere fungal communities differed from nonurban to urban environments, in parallel with a general enrichment of foliar macronutrients in urban trees. In addition to changes in air quality and leaf composition, the differences in tree leaf bacterial community composition that we observed could also have been driven by the increased stress level experienced by urban trees or by changes in some key leaf traits induced by the urban environment.

Considering that the urban gradient index used in this work is based on data representing a combination of parameters such as population density, built-up areas, road access, landscape use, and electric power infrastructure from nine public data sets (31), future studies of urban leaf microbiome dynamics should aim to disentangle the relative influences of these parameters. Anthropogenic stresses on urban trees dramatically reduce the life span of trees to a few years (33, 64, 65). Understanding how tree-microbe interactions potentially accentuate or reduce these stresses is key to helping to improve the management of the urban forest, especially in the face of a changing climate, intensified world trade, and increased pest and disease abundance in urban areas. Urban trees often suffer from limited access to water, which has strong consequences for growth by affecting many processes linked to photosynthesis such as respiration, protein synthesis, and secondary carbohydrate metabolism (66, 67) and therefore also for leaf gas exchanges (68). Another factor with consequences for the leaf microhabitat is heavy metal deposition on leaves driven by air contamination. If water availability influences the potential interactions between the host and microbes at the leaf surface, the deposition of heavy metals (i.e., Cd, Pb, Zn, and Ni) on the leaf in urban environments (69) can also influence the growth capacity of phyllosphere microbes following their metabolic pathways. This hypothesis also concurs with our results demonstrating a greater number of indicators (OTUs) associated with the highest level of urban intensity. Future studies could test the hypothesis that increased anthropogenic stresses select for specific OTUs with higher resistance to stress. Therefore, human population density, local temperature, access to water, air contamination, physical procedures performed on trees, and leaf urban microconditions are all potential drivers of the changes in tree leaf microbiome.

Several studies have suggested that urban areas retain only a limited quantity of biodiversity (70–72), whereas other empirical studies have suggested that these areas could support diverse assemblies of organisms (73, 74). Our results show that, regarding the tree leaf bacterial communities, diversity increases with urban intensity, which allows us to reject our second hypothesis. Although this report cannot provide a mechanistic explanation for this augmentation, we hypothesize that the increased leaf bacterial diversity at high urban intensity could be linked to the local human population density and the heavy metal deposition. The local human population contributes to the location-specific pool of bacterial colonizers, which act as a source for tree leaf microbiomes (75). In parallel, the local air contamination could modify drastically phyllosphere microhabitat growth conditions and toxicity, therefore impacting the microbe-microbe interactions in the phyllosphere.

Increasing levels of anthropogenic pressures, including land use changes, biogeochemical changes, global warming, and exotic species invasion, cause an augmentation of tree stress and a corresponding diminution of longevity and productivity (52–54). Here, we show that the degree of tree isolation, as well as its interactions with host species identity or with urban intensity or with both variables, participates in driving tree phyllosphere community structure (Table 1). In 1983, Baldwin and Schultz (76) and Rhoades (77) revealed that conspecific trees had the capacity to influence the gene expression of neighboring trees through the emission of volatile organic compounds (VOC). These studies revealed that plants possess key mechanisms to “communicate” with each other when attacked by pathogens or pests, suggesting that trees can potentially benefit from proximity to other trees. However, little is known about the influence of the tree leaf microbiome on tree VOC emissions. Urban trees are submitted to multiple anthropogenic stresses of different lengths and intensities, leading to photosynthetic biomass loss and tree lesions (51) which could impact retroactively their interactions with leaf microbiota. In combination with previous work on a tree diversity experiment (28), our results highlight the potential role of the plant neighborhood in driving its leaf microbial community structure. The significant interactions of host tree species with urban intensity or with the degree of isolation or with both factors suggest that each tree species leaf microbiome was influenced differently by these environmental factors (Table 1). Further longitudinal experiments are needed to follow the temporal changes in tree-associated microbiota transplanted from natural to urban environments that occur as the host tree adapts to its new abiotic and biotic conditions. Future studies of the tree microbiome should also aim at describing how the change in the phyllosphere microbiome from natural to urban environments affects tree host fitness. Because of the size and longevity of trees, alternative greenhouse experiments with model organisms such as *Arabidopsis thaliana* could offer a great opportunity to disentangle the influences of the urban environment (i.e., temperature, air contamination, access to water, and physical lesions) and leaf microbial communities. Future experiments developing a “germfree” or “microbiome-controlled” plant growth system in greenhouses will allow researchers to separate the relative influences of the environment and the host microbiome.

Our results support previous findings showing that rural and urban microbial communities differ in composition (6, 7, 78). The proportion of green spaces and species diversity have been suggested as potential drivers of these natural environment-urban environment differences in community composition (8), but our work shows that the plant-associated microbiota *per se* is different from what is usually found in the natural environment as described in previous work in the natural temperate forest (26, 27). Urban abiotic and biotic conditions linked directly and indirectly to human actions are potential drivers of the changes in leaf microbial community structure. Therefore, future studies comparing the relative influences of the increased stress, the sources of microbial input, and the host capacity to select their microbiota in urban settings on the plant-associated microbiome are required to identify clearly the causes of this shift in the urban plant microbiome. In particular, future greenhouse experiments characterizing the influence of physical lesion, nutrient deficiency, water limitation, and air contamination on the plant microbiome and plant fitness will provide key information to support the development of the management of the urban microbiome. Such studies have the potential of identifying the most effective interventions to manage the urban plant microbiome (i.e., increasing plant diversity, increasing plant cover, reducing heat islands, reducing air contamination, introducing specific plant species). Although this work focused on bacterial communities, leaf-inhabiting fungi, yeasts, bacteriophages, and small eukaryotes could be impacted by the higher intensity of anthropogenic pressures but could also interact with tree leaf bacterial communities. Therefore, upcoming studies of the urban plant microbiome focusing on these organisms will definitely provide crucial information to the field, especially in considering the tree phyllosphere as a vector for the airborne microbiome. In addition, including an estimation of the leaf microbial load would allow future work to directly assess the quantitative effect of increased anthropogenic pressures on the phyllosphere microbiome. Finally, our work here also highlights the importance of future studies aimed at understanding the impact of this microbial enrichment in tree leaf microbial communities on host tree health through pathogen infections and pest attacks.

## MATERIALS AND METHODS

### Study sites.

The three study sites are located along an urban intensity gradient on the Island of Montreal, Canada, characterized by a cold and humid continental climate with temperate summer, and were classified as follows: Pierrefonds (45°27′26″N; 73°53′14″W) represented low urban intensity; Ahuntsic (45°33′22″N; 73°39′49″W) represented medium intensity; and Mont-Royal (45°31′32″N; 73°34′00″W) represented high intensity (Fig. 1). We assessed the urban intensity of the locations of the sampled trees based on a composite index of human influence (IHI) as described by Nock et al. (32). This index incorporates information on human infrastructures and presence, movements, landscape use, and electric infrastructure (31) to estimate the footprint of human influence. The IHI of the trees sampled ranged from 38 to 60 in function with respect to site identity and tree isolation.

### Bacterial community collection.

All samples were acquired on 31 July 2014. For each randomly chosen mature tree (at least 20 cm in diameter), we clipped 50 to 100 g of shade leaves at midcanopy height (1 to 2 m above the bottom of the tree’s canopy) into sterile roll bags with surface-sterilized shears. For bacterial community collection and amplification, we used the protocols described by Kembel et al. (20). We collected microbial communities from the leaf surface by agitating the samples in a diluted Redford buffer solution and then resuspended cells in 500 μl of PowerSoil bead solution (MoBio, Carlsbad, CA). We extracted DNA from isolated cells using a PowerSoil kit according to the manufacturer’s instructions and stored the DNA at −80°C.

### DNA library preparation and sequencing.

Samples were amplified using a one-step PCR step and normalized with primers designed to attach a 12-bp barcode and Illumina adaptor sequence to the fragments during PCR (79). For all samples, we used chloroplast-excluding primers targeting the V5-V6 region (799F and 1115R [24]) of the 16S rRNA gene. These primers contained a heterogeneity spacer along with the Illumina linker sequence (forward [799F], 5′-CAAGCAGAAGACGGCATACGAGATGTGACTGGAGTTCAGACGTGTGCTCTTCCGATCTxxxxxxxxxxxx-HS-AACMGGATTAGATACCCKG-3′; reverse [1115R], 5′-AATGATACGGCGACCACCGAGATCTACACTCTTTCCCTACACGACGCTCTTCCGATCTxxxxxxxxxxxx-HS-AGGGTTGCGCTCGTTG-3′) where x represents barcode nucleotides and HS represents a 0-to-7-bp heterogeneity spacer. Each sample was submitted to a single PCR procedure using a 25-µl mixture containing 5 µl 5× HF buffer (Thermo Scientific), 0.5 µl deoxynucleoside triphosphates (dNTPs) (10 µM), 0.5 µl forward primer (10 µM), 0.5 µl reverse primer (10 µM), 0.75 µl dimethyl sulfoxide (DMSO), 0.25 µl Phusion HotStart II polymerase (Thermo Scientific), 1 µl DNA, and 16.5 µl molecular-grade water. The reaction was performed using 30 s of initial denaturation at 98°C followed by 35 cycles of 15 s at 98°C, 30 s at 60°C, and 30 s at 72°C, with a final 10-min elongation at 72°C. The resulting products of natural forest samples were cleaned using a MoBio UltraClean PCR cleanup kit. We isolated an ~445-bp fragment by electrophoresis in a 2% agarose gel and recovered DNA with a MoBio GelSpin kit. We prepared multiplexed 16S libraries by mixing equimolar concentrations of DNA and sequenced the DNA library using Illumina MiSeq 250-bp paired-end sequencing at Genome Quebec. The urban samples were processed with an Invitrogen Sequalprep PCR cleanup and normalization kit (Frederick, MD) and were then pooled with equal concentrations and sequenced on an Illumina MiSeq platform at the University of Montreal. To ensure the quality of our sequencing, we included our negative and positive controls in the sequencing runs.

We processed the raw sequence data with PEAR (80) and QIIME (81) pipelines to merge paired-end sequences to a single sequence of a length of approximately 350 bp, to eliminate low-quality sequences (mean quality score of <30 or with any series of five bases with a quality score of <30), and to demultiplex sequences into samples. We eliminated chimeric sequences using the Uclust and Usearch algorithms (82). We then binned the remaining sequences into operational taxonomic units (OTUs) at a 97% sequence similarity cutoff level using the Uclust algorithm (82). We determined the taxonomic identity of each OTU using the BLAST algorithm and the Greengenes database (83) as implemented in QIIME (81).

### Statistical analyses.

To exclude any spurious OTUs that might have been created by PCR or sequencing errors, we filtered the OTUs to remove those that were represented by fewer than 5 sequences. Rarefactions and analyses were repeated 100 times and showed no qualitative differences across iterations. Therefore, we present the result of a single random iteration. We performed analyses with the ape (84), picante (85), phyloseq (86), ggtern (87), and vegan (88) packages in R (89).

### Data availability.

The data sets supporting the conclusions of this article are available in the Urban Tree phyllosphere Microbiome project repository on Figshare at https://figshare.com/s/07342fc588afdf8d1499 for the R code; https://figshare.com/s/16b8c237fe9b46d3da9b for the metadata; and, finally, https://figshare.com/s/af205b286c6f88f4b1f1 for the 16S RNA sequences.
