# Supplementary material for: Tree Leaf Bacterial Community Structure and Diversity Differ along a Gradient of Urban Intensity
Source: mSystems. 2017 Dec 5;2(6):e00087-17. doi: 10.1128/mSystems.00087-17 (PMC5715107; doi:10.1128/mSystems.00087-17)
Supplement: TABLE S1 [file sys006172158st1.docx]

**Table S1.**

| **Site** | **Urban gradient** | **Tree isolation** | **Urban gradient** | |
| --- | --- | --- | --- | --- |
|  |  |  | **#species** | **# samples** |
| Pierrefonds | Low  38-42 | Street | 7 | 21 |
|  |  | Park |  | 21 |
| Ahuntsic | Med  50-60 | Street | 7 | 21 |
|  |  | Park |  | 21 |
| Mont-Royal | High  50-60 | Street | 7 | 21 |
|  |  | Park |  | 21 |
