# Supplementary material for: Tree Leaf Bacterial Community Structure and Diversity Differ along a Gradient of Urban Intensity
Source: mSystems. 2017 Dec 5;2(6):e00087-17. doi: 10.1128/mSystems.00087-17 (PMC5715107; doi:10.1128/mSystems.00087-17)
Supplement: TABLE S2 [file sys006172158st2.docx]

**Table S2.**

a)

b)

c)

| **CLASS** | **ORDER** | **FAMILY** | **GENERA** | **SPECIES** | **IND. SCORE** | **P-VALUE** |
| --- | --- | --- | --- | --- | --- | --- |
| Actinobacteria | Actinomycetales | Kineosporiaceae | NA | NA | 0.34 | 0.031 |
|  |  | Microbacteriaceae | Frondihabitans | cladoniiphilus | 0.32 | 0.001 |
|  |  | Nocardioidaceae | Friedmanniella | NA | 0.37 | 0.001 |
|  |  | NA | NA | NA | 0.42 | 0.008 |
| Alphaproteobacteria | Rhizobiales | Beijerinckiaceae | NA | NA | 0.31 | 0.04 |
|  | Rhodobacterales | Rhodobacteraceae | Paracoccus | NA | 0.38 | 0.046 |
|  |  |  | Rubellimicrobium | NA | 0.46 | 0.022 |
|  | Rhodospirillales | Acetobacteraceae | Roseomonas | NA | 0.41 | 0.014 |
|  |  |  |  | NA | 0.31 | 0.01 |
|  |  |  | NA | NA | 0.54 | 0.001 |
|  |  |  | NA | NA | 0.39 | 0.019 |
|  |  |  | NA | NA | 0.32 | 0.015 |
|  |  | Rhodospirillaceae | Skermanella | NA | 0.36 | 0.021 |
|  | Sphingomonadales | Sphingomonadaceae | Sphingomonas | asaccharolytica | 0.39 | 0.001 |
| Betaproteobacteria | Burkholderiales | NA | NA | NA | 0.43 | 0.002 |
| Cytophagia | Cytophagales | Cytophagaceae | Adhaeribacter | NA | 0.37 | 0.003 |
|  |  |  |  | NA | 0.35 | 0.001 |
|  |  |  | Hymenobacter | NA | 0.59 | 0.001 |
|  |  |  |  | NA | 0.42 | 0.001 |
|  |  |  |  | NA | 0.40 | 0.001 |
|  |  |  |  | NA | 0.40 | 0.001 |
|  |  |  |  | NA | 0.34 | 0.009 |
|  |  |  |  | NA | 0.32 | 0.026 |
| Deinococci | Deinococcales | Deinococcaceae | Deinococcus | aquatilis | 0.67 | 0.001 |
|  |  |  |  | NA | 0.73 | 0.001 |
|  |  |  |  | NA | 0.66 | 0.001 |
|  |  |  |  | NA | 0.60 | 0.001 |
|  |  |  |  | NA | 0.59 | 0.001 |
|  |  |  |  | NA | 0.53 | 0.001 |
|  |  |  |  | NA | 0.48 | 0.001 |
|  |  |  |  | NA | 0.42 | 0.001 |
|  |  |  |  | NA | 0.32 | 0.004 |
| Fimbriimonadia | Fimbriimonadales | Fimbriimonadaceae | Fimbriimonas | NA | 0.41 | 0.028 |
| Gammaproteobacteria | Enterobacteriales | Enterobacteriaceae | NA | NA | 0.38 | 0.001 |
|  |  |  | NA | NA | 0.32 | 0.001 |
| Saprospirae | Saprospirales | Chitinophagaceae | NA | NA | 0.46 | 0.03 |
|  |  |  | NA | NA | 0.38 | 0.001 |
